# Dental artifacts in the head and neck region: implications for Dixon-based attenuation correction in PET/MR

**DOI:** 10.1186/s40658-015-0112-5

**Published:** 2015-03-11

**Authors:** Claes N Ladefoged, Adam E Hansen, Sune H Keller, Barbara M Fischer, Jacob H Rasmussen, Ian Law, Andreas Kjær, Liselotte Højgaard, Francois Lauze, Thomas Beyer, Flemming L Andersen

**Affiliations:** Department of Clinical Physiology, Nuclear Medicine and PET, Rigshospitalet, University of Copenhagen, Blegdamsvej 9, 2100 Copenhagen East, Denmark; Department of Oncology, Rigshospitalet, University of Copenhagen, Blegdamsvej 9, 2100 Copenhagen East, Denmark; Department of Computer Science, University of Copenhagen, Universitetsparken 5, 2100 Copenhagen East, Denmark; Centre for Medical Physics and Biomedical Engineering, Medical University of Vienna, Waehringer Guertel 18-20/4L, Vienna, A-1090 Austria

**Keywords:** PET/MRI, Attenuation correction, Metal artifacts, Quantification, Inpainting

## Abstract

**Background:**

In the absence of CT or traditional transmission sources in combined clinical positron emission tomography/magnetic resonance (PET/MR) systems, MR images are used for MR-based attenuation correction (MR-AC). The susceptibility effects due to metal implants challenge MR-AC in the neck region of patients with dental implants. The purpose of this study was to assess the frequency and magnitude of subsequent PET image distortions following MR-AC.

**Methods:**

A total of 148 PET/MR patients with clear visual signal voids on the attenuation map in the dental region were included in this study. Patients were injected with [^18^F]-FDG, [^11^C]-PiB, [^18^F]-FET, or [^64^Cu]-DOTATATE. The PET/MR data were acquired over a single-bed position of 25.8 cm covering the head and neck. MR-AC was based on either standard MR-AC_DIXON_ or MR-AC_INPAINTED_ where the susceptibility-induced signal voids were substituted with soft tissue information. Our inpainting algorithm delineates the outer contour of signal voids breaching the anatomical volume using the non-attenuation-corrected PET image and classifies the inner air regions based on an aligned template of likely dental artifact areas. The reconstructed PET images were evaluated visually and quantitatively using regions of interests in reference regions. The volume of the artifacts and the computed relative differences in mean and max standardized uptake value (SUV) between the two PET images are reported.

**Results:**

The MR-based volume of the susceptibility-induced signal voids on the MR-AC attenuation maps was between 1.6 and 520.8 mL. The corresponding/resulting bias of the reconstructed tracer distribution was localized mainly in the area of the signal void. The mean and maximum SUVs averaged across all patients increased after inpainting by 52% (± 11%) and 28% (± 11%), respectively, in the corrected region. SUV underestimation decreased with the distance to the signal void and correlated with the volume of the susceptibility artifact on the MR-AC attenuation map.

**Conclusions:**

Metallic dental work may cause severe MR signal voids. The resulting PET/MR artifacts may exceed the actual volume of the dental fillings. The subsequent bias in PET is severe in regions in and near the signal voids and may affect the conspicuity of lesions in the mandibular region.

**Electronic supplementary material:**

The online version of this article (doi:10.1186/s40658-015-0112-5) contains supplementary material, which is available to authorized users.

## Background

PET is a powerful and accurate diagnostic imaging method for the assessment of oncology patients [1]. Combined positron emission tomography/computed tomography (PET/CT) has been shown to provide intrinsically aligned functional and anatomical image information from a single patient's examination [2]. Combined PET/CT imaging offers the additional advantage of using the CT images for noise-limited attenuation correction (CT-based attenuation correction, CT-AC) [3]. The accuracy of the CT-AC is limited by beam-hardening artifacts caused by the significantly higher photon absorption from high-Z materials compared with low-Z materials (e.g., soft tissues) at CT energies [4].

Recently, combined PET/MR systems have become available to clinical users [5] and found to be of value in cancer imaging [6–9]. Combined PET/MR helps present PET information in the context of sequentially or simultaneously acquired MR data. However, in the absence of CT or traditional transmission sources in PET/MR systems, the MR images are also used for MR-based AC (MR-AC) [10]. The susceptibility effects arising from metal implants may challenge the accuracy of MR-AC [11–14]. This leads to signal voids translating into artifactual attenuation values equal to air in the proximity of the implants. This could affect response assessment where correct attenuation correction is required in order to allow for quantitative evaluation [15]. More specifically, there will be an underestimation of PET activity after attenuation correction due to this assignment of zero attenuation in the artifactual MR-based attenuation maps. Reversely, the scatter will be undercorrected leading to an overestimation of PET activity. These two effects will have opposite effects, although the lack of attenuation will likely be the dominant effect in areas of the susceptibility artifacts.

One particular common metal artifact in MR, also well known from CT and PET/CT, relates to dental fillings and metal braces [11,13]. These artifacts have implications for, among others, tumor imaging in the head and neck region. Head and neck cancer ranks among the ten most common malignant diseases [16]. Patients suffering from head and neck cancer may benefit from PET/MRI-based workup, since MR provides a better soft tissue contrast than CT in this region [17], which has made it the modality of choice for imaging tumors of the oral cavity [18] and pharynx [19].

The aim of this study was to assess the prevalence and magnitude of regional and absolute bias in PET activity as a result of ignoring susceptibility artifacts in the dental region during MR-AC of combined PET/MR images of the head and head/neck. The clinical data from neurology and oncology patients with dental artifacts of different sizes were employed.

## Methods

### Patients

Data sets were gathered retrospectively from the patient cohort imaged with the combined PET/MR (Siemens Biograph mMR, Siemens Healthcare, Erlangen, Germany) at our institution between February 2012 and September 2013. During this period, 339 patients presented with a clinical indication for a PET/MR examination of the brain or the head and neck region. The total number of patients showing artifacts in the form of signal voids on MR-AC attenuation maps was counted and selected for manual region of interest (ROI) delineation and analysis. The studies were approved by the local Ethics committee and all patients gave informed consent (approval no. H-3-2012-072, SJ-214/28.464).

### Imaging protocol

Patients were injected with [^18^F]-FDG, [^11^C]-Pittsburgh compound B (PiB), O-2-([^18^F]-fluoroethyl)-L-tyrosine (FET), [^64^Cu]-DOTATATE, or [^68^Ga]-DOTATOC (see Table 1). Patients were positioned on the fully integrated PET/MR system (Biograph mMR, Siemens Healthcare) [20]. All patients were positioned head-first, with their arms down, and data was acquired over a single-bed position of 25.8 cm covering the head and neck. Standard MR-based attenuation maps (MR-AC_DIXON_) were derived using the Dixon VIBE sequence [21]. For the purpose of this study, the PET data from the PET/MR acquisition were corrected for attenuation using MR-AC_DIXON_ as well as an attenuation map where dental artifacts had been automatically corrected (MR-AC_INPAINTED_). The PET images were reconstructed with and without MR-AC using 3D ordinary Poisson-ordered subset expectation maximization (OP-OSEM) (three iterations, 21 subsets, 4-mm Gaussian post filtering) on 344×344 matrices (2.1 × 2.1 × 2.0 mm voxels) into the resulting images PET_DIXON_ and PET_INPAINTED_. The Dixon-water image and the MR-AC attenuation maps were reconstructed on 192 × 126 × 128 matrices (2.6 × 2.6 × 3.1 mm^3^ voxels). For the purpose of ROI delineation, a sagittal T1-weighted (T1w) MPRAGE was used, with a matrix size of 512 × 512 × 192 (0.5 × 0.5 × 1 mm^3^ voxels).

**Table 1:** **PET tracer information for all 144 patients included in this study**

**Tracer**	**Number of patients**	**Dose [MBq ± std]**	**Median post injection time [min] (range)**
[^18^F]-FDG (brain)	75	203 ± 16	51 (35 to 141)
[^18^F]-FDG (head/neck)	25	358 ± 59	129 (100 to 181)
[^11^C]-PiB	20	447 ± 151	43 (36 to 86)
[^18^F]-FET	19	211 ± 11	22 (0 to 89)
[^64^Cu]-DOTATATE	4	197 ± 18	83 (75 to 88)
[^68^Ga]-DOTATOC	1	144	62

### Corrected attenuation map: inpainting

**Figure 1:**
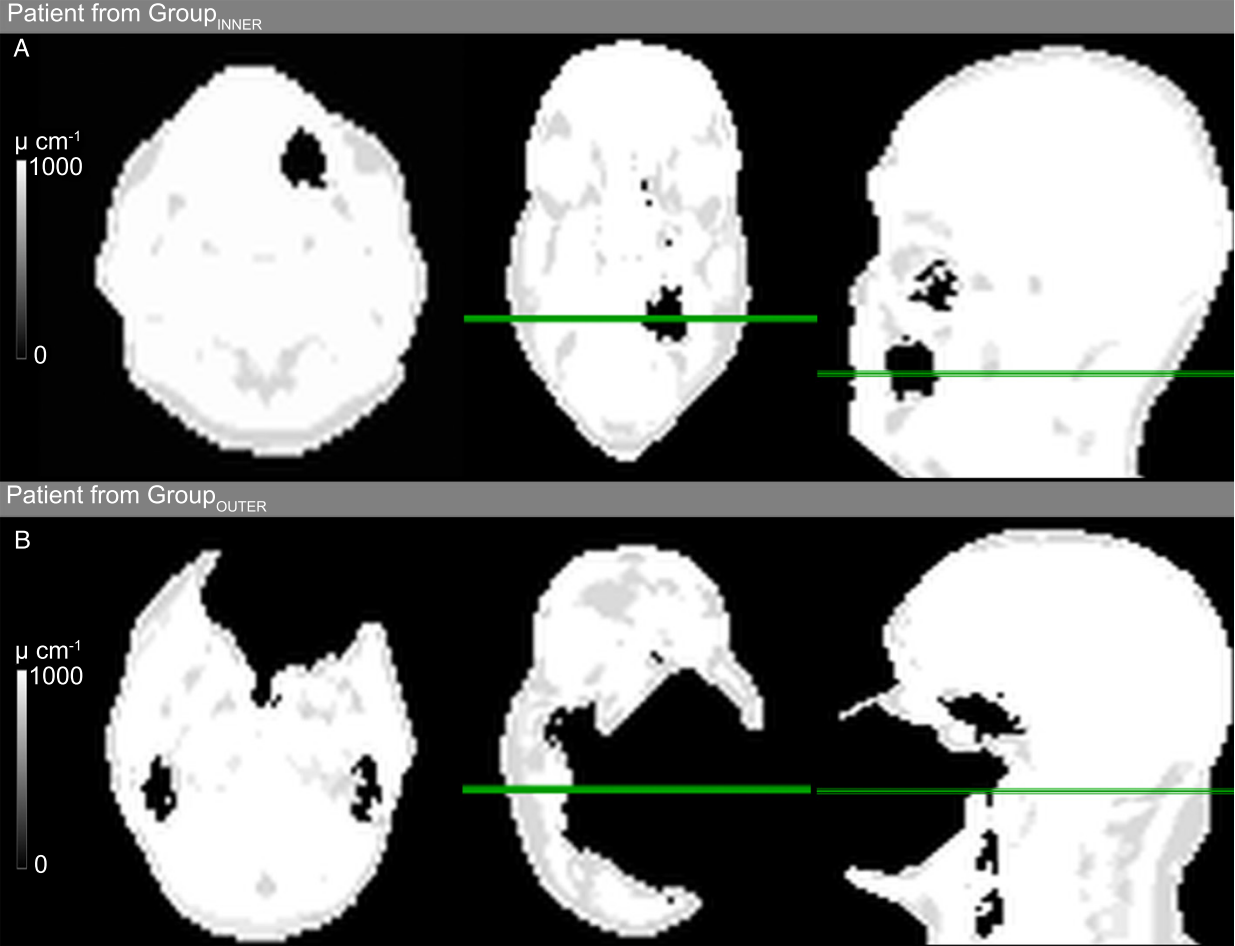
**Separation of patients by semi-automatic investigations of the connected neighborhoods for each artifact. (A)** Patient from Group_INNER_ where the artifact is fully enclosed by the anatomical surface. **(B)** Patient from Group_OUTER_ where the artifact breaches the anatomical surface and is artificially connected to the background. From left to right: transaxial view, coronal view and sagittal view of MR-AC_DIXON_ attenuation maps.

**Figure 2:**
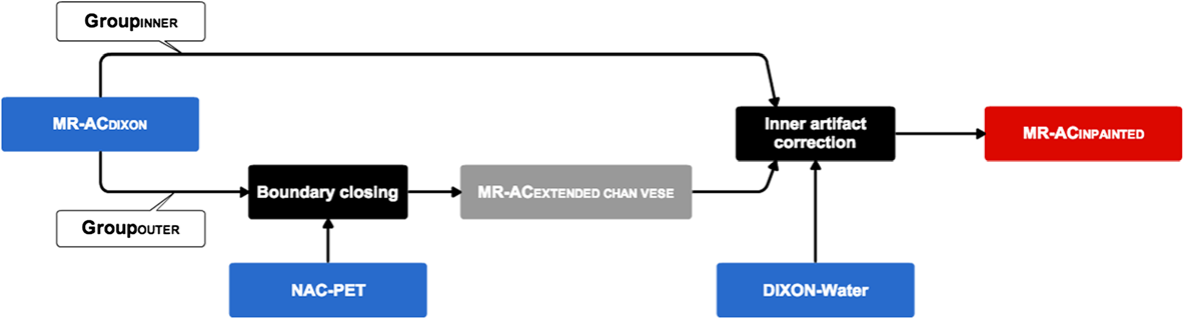
**Workflow of algorithm.** (1) The boundary is found and closed for the patients in Group_OUTER_ by the use of the NAC-PET image and MR-AC_DIXON_. (2) For both groups, the patients' Dixon-water image is aligned to an atlas, and the air regions within the anatomical surface and overlapping with the mask drawn on the atlas will be filled. The resulting image is denoted MR-AC_INPAINTED_.

Artifacts that exceeded the anatomical boundary and those that did not were corrected differently. Figure 1 illustrates the separation of patients by semi-automatic investigations of the connected neighborhoods for each artifact into two groups. In Group_INNER_, the signal voids were enclosed within the anatomical boundary (*n* = 76, Figure 1A), and in Group_OUTER_, the signal voids exceeded the anatomical boundary (*n* = 72, Figure 1B). The steps of the algorithm [22] are illustrated in Figure 2. In short, the method initially delineates the outer contour of the artifacts in Group_OUTER_ by a previously published algorithm [22] based on the original level set segmentation algorithm by Chan and Vese [23]. The algorithm is extended such that it punishes deviations between MR-AC_DIXON_ and the non-attenuation-corrected (NAC)-PET image. In areas with a signal void on MR-AC_DIXON_, the outer contour follows the area on NAC-PET, representing the edge, and closes any signal void breaching the boundary. The signal voids now closed by the found boundary were filled with a value representing the attenuation of soft tissue (0.1 cm^−1^). In areas without signal voids, the contour follows MR-AC_DIXON_. All parameters used in the segmentation were automatically determined by inspecting the PET signal outside MR-AC_DIXON_. No preprocessing was performed on the patients in Group_INNER_, as the anatomical boundary is not breached by the signal void.

Then for both groups, the method attempts to correct the signal voids within the anatomical boundary in a two-step process. First, the Dixon-water image of the patient was aligned with an atlas where the oral cavity had been masked. The atlas represents the Dixon-water image averaged across 30 patients without dental artifacts. By investigating the area where no patients had signal voids, it was possible to identify the boundaries of the oral cavity. Second, all signal void regions overlapping with the mask were filled with a value representing the attenuation of soft tissue (0.1 cm^−1^), thereby assuming that noticeable signal voids in the dental region result from metallic dental implants only. Third, all partly filled signal voids were inspected automatically. Those filled more than 80% of the total volume were filled completely, and those filled less than 10% were considered an error and therefore reversed. This was done to complete artifact voids extending outside the oral cavity mask or to compensate for smaller errors in the registration to the atlas. The high threshold of 80% was chosen such that the sinuses did not get filled if these were connected to the dental artifact regions.

### Image analysis

All MR-AC_INPAINTED_ attenuation maps were visually inspected to ensure that the inpainting was performed correctly. For patients where the metal-implant-induced signal void were connected to the sinuses, we used the T1w MR image and an aligned CT image when available, to inspect the accuracy of the correction. Visual and quantitative analyses of the reconstructed PET images from the PET/MR examinations following MR-AC with MR-AC_DIXON_ and MR-AC_INPAINTED_ were performed. To assess the potential effects in the dental region and further away, four ROIs were delineated manually in reference regions (Additional file 1: Figure S1) seen on transaxial T1w images (tongue, lower tongue, and masticatory muscle; the latter a combination of left and right sub-ROIs) and PET_INPAINTED_ images (cerebellum) using the Mirada software (version XD3.4, Mirada Medical Ltd, New Road, Oxford, UK). If the patient did not have a T1w image due to older imaging protocols (28/148 patients), ROIs were defined on the Dixon-water image instead. In cases where the lack of MR signal prevented ROI delineation, the ROIs were defined on PET_INPAINTED_ instead. For each patient and image reconstruction, the mean PET activity concentration was calculated for the fully inpainted area as well as for each of the four ROIs following correction. The size of each of the artifacts was measured. We also calculated the relative differences (mean and max):

1 $$ {\varepsilon}_x^{rel} = \frac{SUV_x^{DIXON}-{SUV}_x^{INPAINTED}}{SUV_x^{INPAINTED}} \times 100\% $$

and the absolute differences:

2 $$ {\varepsilon}_x^{abs} = {SUV}_x^{DIXON}-{SUV}_x^{INPAINTED}, $$

for all patients and ROIs, where *x* denotes mean or max SUV of PET_DIXON_ and PET_INPAINTED_, respectively.

The Kolmogorov-Smirnov test was performed to test for a normal distribution of SUV_MEAN_ values. Since data were not normally distributed, a Wilcoxon signed-rank test for matched pairs was applied to test for differences in SUV_MEAN_ between ROIs in PET_DIXON_ and PET_INPAINTED_; *p* < 0.05 was considered to be statistically significant. Furthermore, to illustrate the effect, we calculated both relative and absolute mean difference images. The overall results for the affected study population as well as the single-patient case studies are presented.

## Results

**Figure 3:**
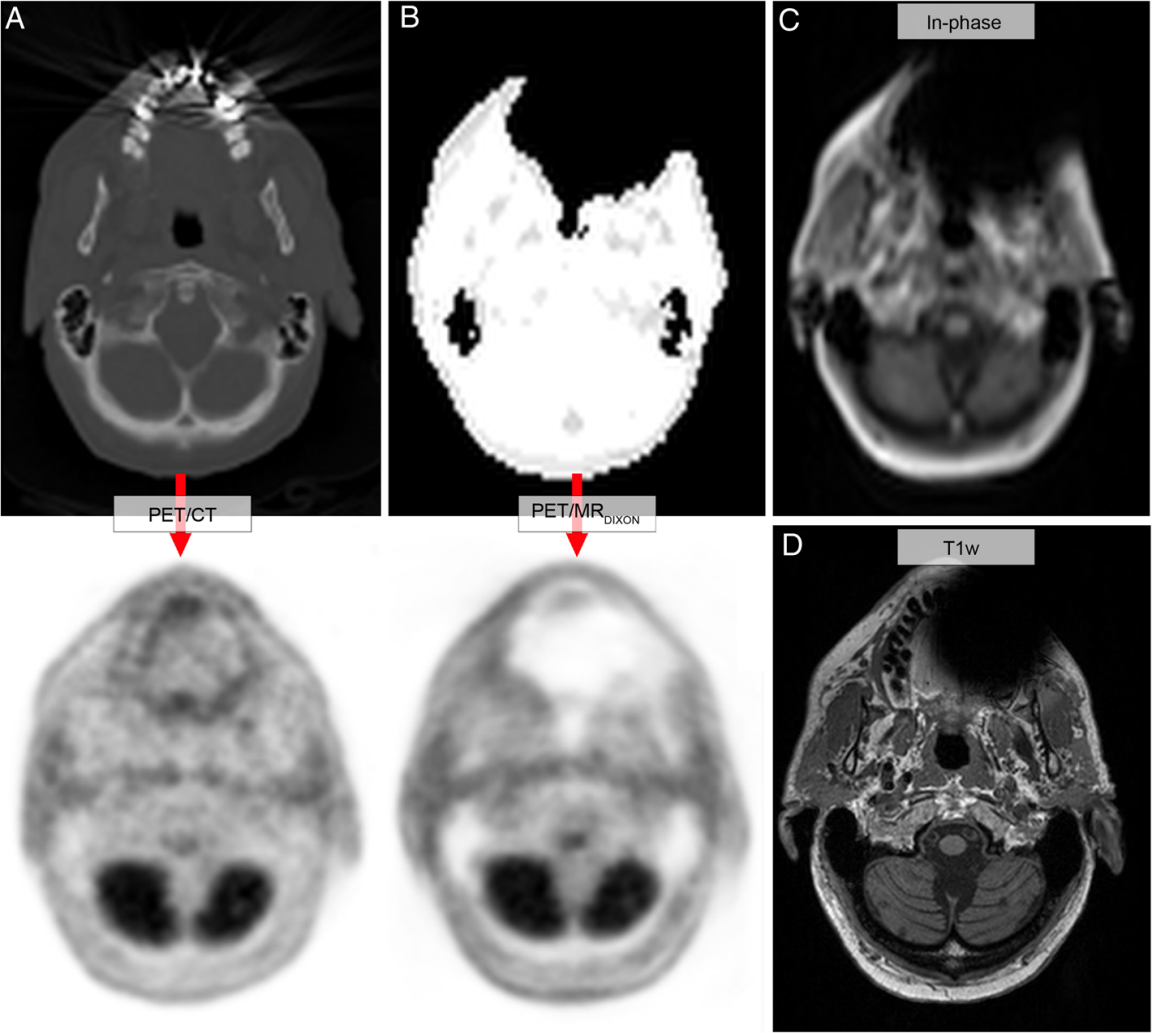
**Transaxial views of a patient from Group**_**OUTER**_ **with dental fillings. (A)** CT and CT-AC-PET (/CT) image. **(B)** MR-based attenuation map and MR-AC-PET (/MR) image. **(C)** In-phase image from Dixon sequence. **(D)** T1w MR image.

**Figure 4:**
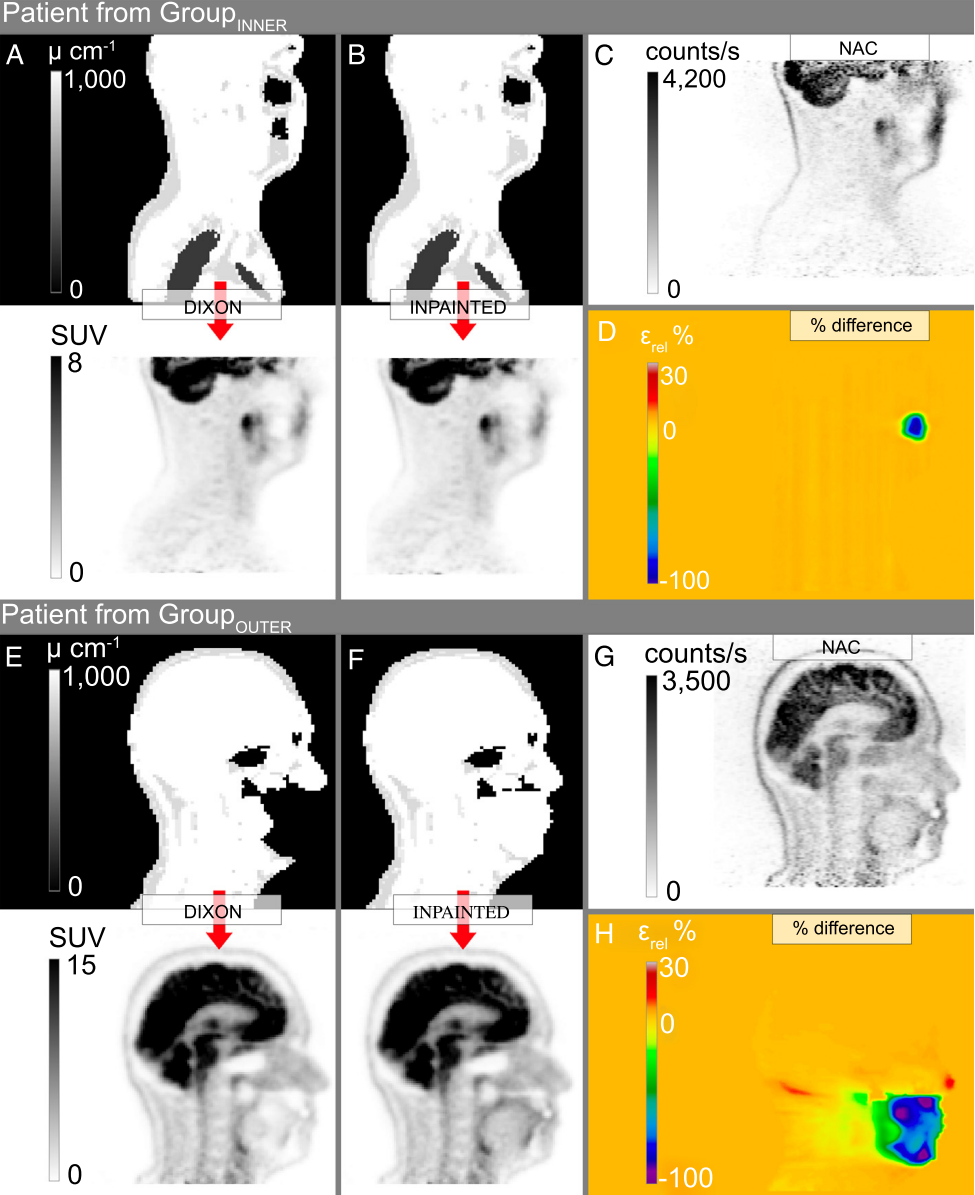
**Representative patients from Group**_**INNER**_ **(A-D) and Group**_**OUTER**_ **(E-H).** Note the recovery of tracer activity in the proximity of the artifact when comparing **(F)** to **(G)**. Panels **(D,H)** show the corresponding relative PET difference images for **(A,B)** in relation to **(B)** and **(E-F)** to **(F)**, respectively.

**Figure 5:**
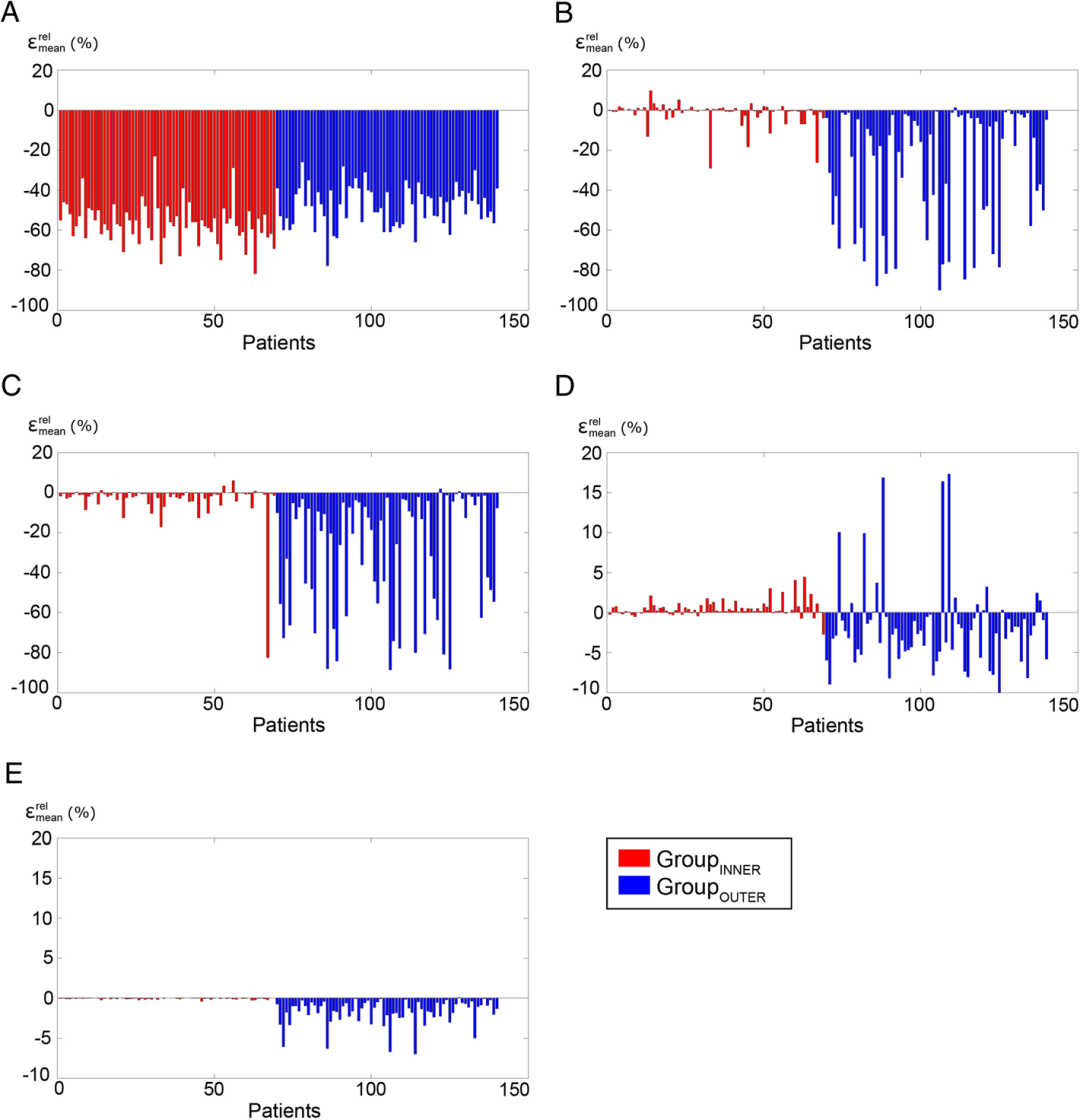
**Relative percent difference between the PET images. (A)** Fully inpainted region. **(B)** Lower tongue. **(C)** Tongue. **(D)** Masticatory muscles. **(E)** Cerebellum. Note the reduced scales on **(D)** and **(E)**. Patient numbering is random within the groups.

**Figure 6:**
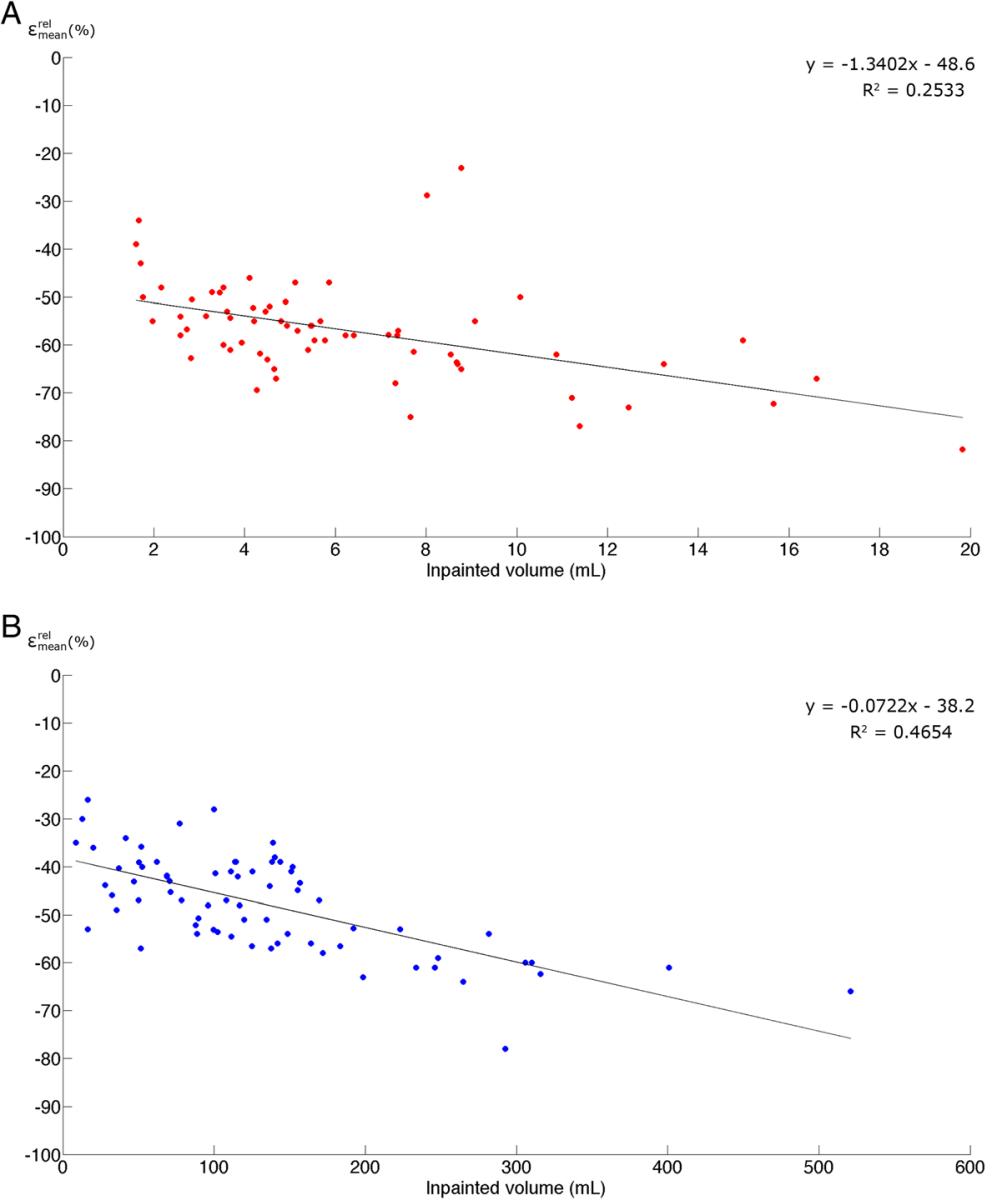
**Volume** **and relative percentage difference on** ***x*** **- and** ***y*** **-axes.** Relative percentage difference shown for the fully inpainted area. **(A)** patients from Group_INNER,_ **(B)** patients from Group_OUTER_.

Of the consecutive cohort of 339 patients, 44% (148) had clear visual signal voids on MR-AC_DIXON_ in the dental region as a result of a metallic implant. In 72/148 patients (49%), the signal voids exceeded the anatomical surface (Figure 1B). Four patients were excluded due to file corruption. The remaining 144 consecutive subjects with signal voids of the dental region were analyzed (Table 1). Our inpainting method was able to automatically correct the signal voids in 98% of these patients (141/144). The correction method failed in the three cases due to abnormal location of the artifact causing it to be classified as part of the maxillary sinus (one patient) and failure to define the outer contour correctly (two FDG patients with unusually small outer artifacts). The assessment was done by visual inspection. Figure 3 shows an example of CT, Dixon, PET, and T1w images for a single patient with a large signal void in the dental region exceeding the anatomical surface (Group_OUTER_). The relative and absolute changes in SUV following our correction method are exemplified for each of the groups in Figure 4. Figure 5 summarizes the relative changes in SUV_MEAN_$\left({\varepsilon}_{\mathrm{mean}}^{\mathrm{rel}}\right)$ for all patients in both groups for the fully inpainted region and the reference regions in the masticatory muscles, cerebellum, tongue, and lower tongue (Additional file 1: Figure S1). The averaged results of the relative changes in SUV_MEAN_$\left({\varepsilon}_{\mathrm{mean}}^{\mathrm{rel}}\right)$ and SUV_MAX_$\left({\varepsilon}_{\max}^{\mathrm{rel}}\right)$ are summarized in Table 2. The ${\varepsilon}_{\mathrm{mean}}^{\mathrm{rel}}$ plotted versus the size of the artifacts for the inpainted area is shown in Figure 6.

**Table 2:** **Averaged relative difference** $\left({\boldsymbol{\varepsilon}}_{\boldsymbol{x}}^{\mathbf{rel}}\right)$ **(Equation** **1** **) of SUV mean and max**

**ROI**	${\boldsymbol{\varepsilon}}_{\mathbf{mean}}^{\mathbf{rel}}$ **(± std)%**	**Range [%]**	${\boldsymbol{\varepsilon}}_{\mathbf{max}}^{\mathbf{rel}}$ **(± std)%**	**Range [%]**
Group_INNER_: internal signal void				
Inpainted area	−57 (10)	−82 to −23	−26 (11)	−49 to −3.3
Lower tongue	−1.6 (6.0)	−29 to 10	−1.0 (4.8)	−23 to 10
Tongue	−3.4 (10)	−82 to 6.1	−1.6 (8.6)	−70 to 4.5
Masticatory muscles	0.5 (1.0)	−2.8 to 4.5	0.3 (1.1)	−3.2 to 4.1
Cerebellum	−0.1 (0.1)	−0.4 to 0.0	0.0 (0.8)	−2.3 to 4.5
Group_OUTER_: signal void affecting exterior body contour				
Inpainted area	−48 (10)	−78 to −26	−24 (10)	−58 to −5.4
Lower tongue	−29 (30)	−90 to 1.4	−23 (26)	−79 to 4.4
Tongue	−30 (29)	−89 to 2.0	−22 (25)	−84 to 3.4
Masticatory muscles	−1.8 (5.4)	−11 to 17	−2.7 (4.6)	−9.9 to 17
Cerebellum	−1.8 (1.6)	−7.0 to 0.1	−1.8 (2.2)	−11 to 2.4

### Patients with internal signal voids

The average size of the artifacts in Group_INNER_ was 6 mL (range, 1.6 to 16.6 mL). The mean SUV difference $\left({\varepsilon}_{\mathrm{mean}}^{\mathrm{rel}}\right)$ averaged across subjects in Group_INNER_ for the ROI covering the fully inpainted area was −57% (range −82% to −23%, Table 2). The tongue region was most affected by inpainting with an average relative change in uptake of −3.4% (range, −82% to 6.1%) for SUV_MEAN_. In the masticatory muscles near the tongue, the bias was predominantly positive, as the average mean change was 0.5% (range, −2.8% to 4.5%), which is in contrast to the other regions where a negative bias was observed. In the regions further away (lower tongue and cerebellum), the mean relative difference were below −1.6% and −0.1%, respectively. In 11 patients, the SUV_MEAN_ in the lower tongue region changed by more than ± 5% (range, −29% to 10%).

### Patients with signal void affecting the exterior body contour

**Figure 7:**
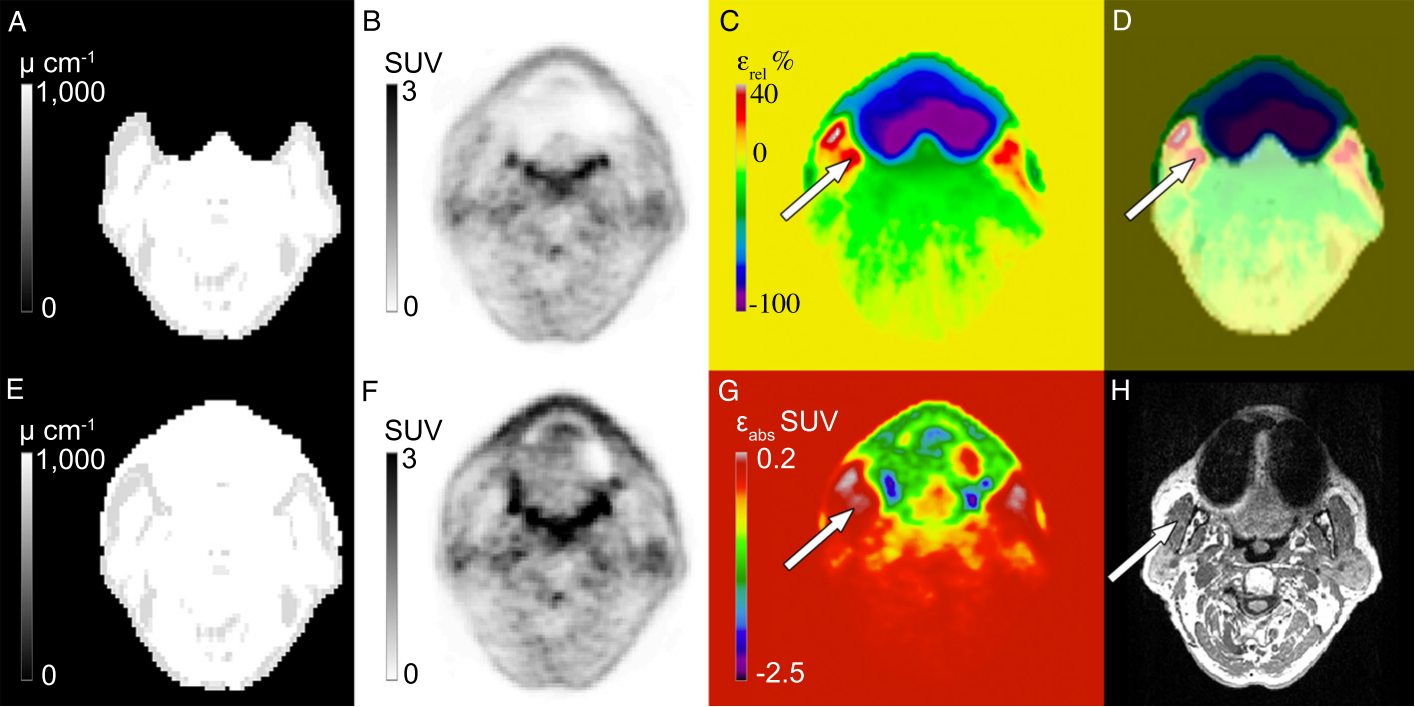
**Images from PET/MR of a patient (Group**_**OUTER**_ **) with large positive bias in regions just outside the artifact. (A)** Original attenuation map. **(E)** Attenuation map after inpainting. **(B,F)** corresponding PET images following MR-AC. Note the recovery of PET signal in the dental region. **(C)** The mean relative difference image (*ε*_rel_) for **(B-F)** in relation to **(F)**. **(G)** Absolute difference image (*ε*_abs_) for **(B)** to **(F)**. Note the positive SUV region in the masticatory muscles. **(D)** A and C fused. **(H)** T1w MR image. Arrows point to the masticatory muscle.

For the Group_OUTER_ patients, the size of the artifacts was much larger with a mean value of 133 mL (range, 8.7 to 520.8 mL). The ${\varepsilon}_{\mathrm{mean}}^{\mathrm{rel}}$ was similar to the results of Group_INNER_ with −48% (range, −78% to −26%, Table 2). The ROI mostly affected by variations in AC was also in the tongue region, with an average mean change of −30% (range, −89% to 2.0%). In the regions near the tongue, e.g., the lower tongue and masticatory muscles, the average mean change was −29% and −1.8%, respectively. It is worth noting that the effects of the correction were also visible in the cerebellum, with an average mean change of −1.8% (range, −7.0% to 0.1%). The maximum difference of SUV_MAX_ was −11%. The bias was mainly negative in the masticatory muscles (mean, −1.8%), but for five of the patients the bias was positive. This concurs with the results from Group_INNER_, however, on a much larger scale (range, 10% to 17%). Figure 7 shows results of a patient (Group_OUTER_) where the bias in the artifact region was negative but positive in the adjacent reference region.

Across Group_INNER_ and Group_OUTER_, the maximum relative change averaged across all subjects was −91% (± 8.3%) (range, −100% to −8.3%) in the fully inpainted region, whereby −100% indicates voxels with a total loss of PET activity. The SUV_MEAN_ values obtained with MR-AC_DIXON_ were statistically different from those obtained with MR-AC_INPAINTED_ in the regions of the tongue, lower tongue, and the fully inpainted region (Wilcoxon, *p* < 0.01).

**Figure 8:**
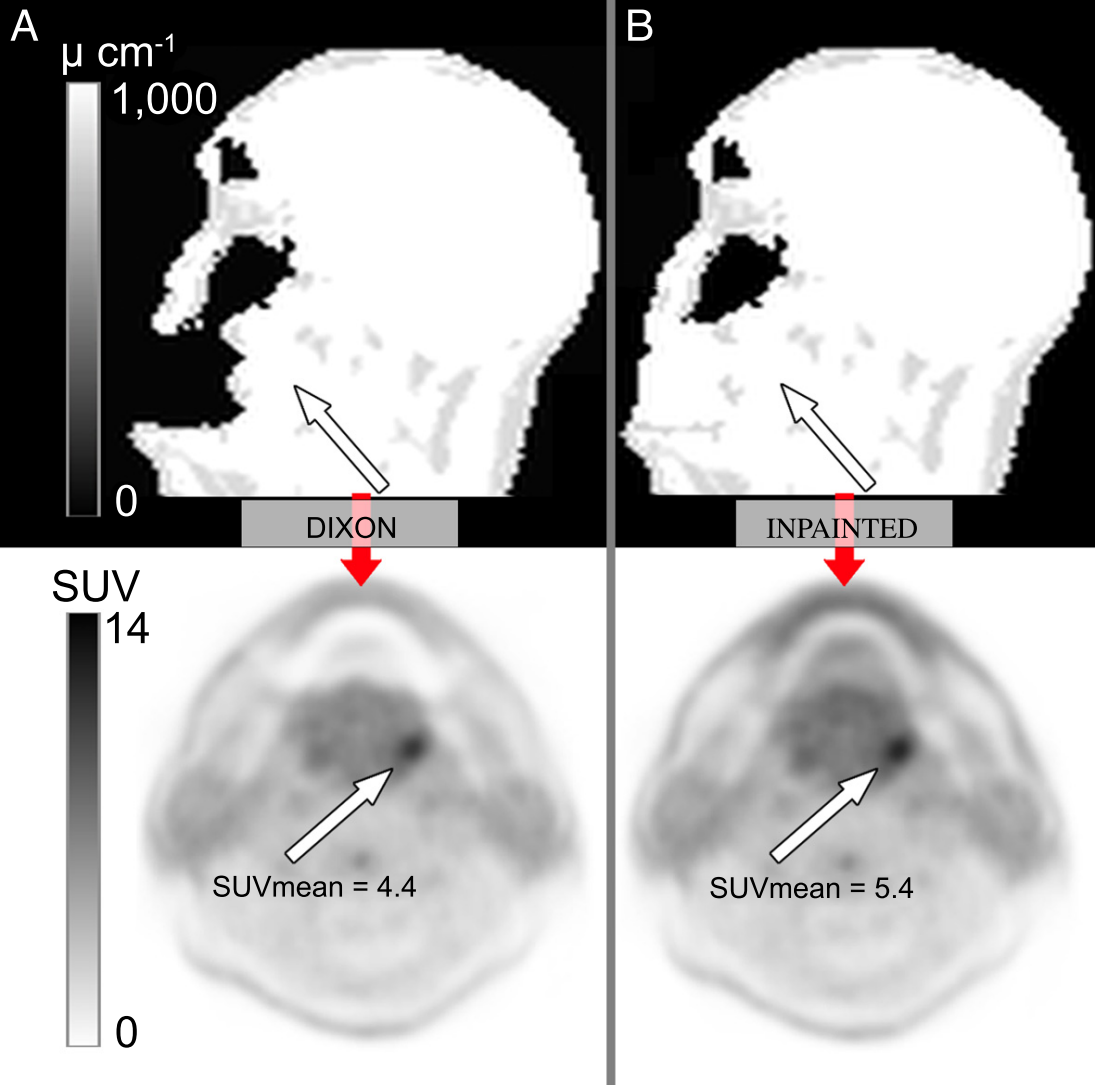
**Effect of dental artifact on soft tissue lesion.** Effect of dental artifact (Group_OUTER_) on soft tissue lesion located to the left, caudally posteriorly in the body of the lingua Dixon **(A)** and inpainted **(B)** with anatomical in top row and PET image in bottom row. Measurements in lesion: mean relative difference, −19%; max relative difference, −18%; mean absolute difference, −1; max absolute difference, −1.7.

An illustrative case is shown in Figure 8. This patient had a 2.9-mL soft tissue lesion located to the left, caudally posterior in the body of the lingua and 25 mm outside the artifact region. The lesion was seen on both PET_DIXON_ and PET_INPAINTED_. The SUV values were, however, markedly higher after correction (${\varepsilon}_{\mathrm{mean}}^{\mathrm{rel}}$ −19% and ${\varepsilon}_{\max}^{\mathrm{rel}}$ −18%; ${\varepsilon}_{\mathrm{mean}}^{\mathrm{abs}}$ −1 and ${\varepsilon}_{\max}^{\mathrm{abs}}$ −1.7).

## Discussion

This study examines the consequences of ignoring metal-implant-induced MR artifacts in the dental region for PET, measured using a combined PET/MR system with standard, vendor-supplied MR attenuation correction. The resulting estimated bias in AC-PET is severe in regions in and near the artifact regions.

Dental artifacts are considered a challenging problem to which no satisfactory solution is yet available [24]. Specialized multispectral MR sequences for imaging near metal [25] help improve the overall quality of the MR images. However, artifacts remain in metal, and the performance of multispectral sequences in the context of AC has not been studied. We note that this type of sequences requires long acquisition times (4 to 10 min has been reported even when using accelerated [26] or off-resonance suppressed schemes [27]), as compared to the 19 s acquisition time for the standard Dixon VIBE.

The effect of ignoring metal artifacts in PET/MR imaging of the head and neck region was studied also by Buchbender [28]. Their study included 19 patients with metal implants, 11 had dental work. The authors measured the SUV_MEAN_ in a volume of interest (VOI) inside all signal voids and found the values to be significantly lower in the artifact region than in the unbiased contralateral reference region (0.05 ± 0.07 versus 0.42 ± 0.22).

So far, 44% of our PET/MR patient population had a metal artifact due to dental implants in the MR-AC_DIXON_ image. Artifacts caused a regional bias in reconstructed activity mainly in the area of the signal void. The effect of the artifact decreased with the distance to the signal void in a non-simple pattern, and biased uptake regions were noticed in regions as far away as the cerebellum. The magnitude of the effect correlated with the actual size of the artifact (Figure 6), and for some artifacts, a complete loss of activity was observed (Figure 4). Of note, such artifacts will be detrimental for the use of PET/MR in a clinical setup where accurate PET measurements are important, such as in the case of response evaluation [15].

Our study included 144 patients and used an automatic algorithm for artifact correction, which is based on a combination of a template of likely artifact areas and a level set segmentation method that delineates the outer contour. The automated correction method was able to substantially reduce the artifacts in 98% of the included patients. The time to correct each attenuation map was about 1 min for the patients in Group_INNER_ and about 40 min for Group_OUTER_ using a standard MacBook Pro (Apple Inc., 1 Infinite Loop, Cupertino, CA, USA) and non-optimized code. It is, therefore, feasible to have images ready for reading with a delay less than an hour. This study included patients injected with five different tracers ([^18^F]-FDG, [^18^F]-FET, [^11^C]-PiB, [^64^Cu]-DOTATATE, and [^68^Ga]-DOTATOC), so the method appears to be robust to the tracer type. The method only makes use of the NAC-PET image and the Dixon-water image to correct for the artifacts; both are readily available in all PET/MR exams, so the method does not require any extra scan time.

For both patient groups, the change in SUV_MEAN_ following correction was largest near the signal voids (tongue, −3.4% Group_INNER_, −30% Group_OUTER_), but for Group_INNER_ the difference decreased in the lower tongue (Table 2 and Figure 5). In regions further away, such as in the cerebellum, no significant differences between the PET values were seen (Figure 4D) as a result of the artifact correction in Group_INNER_. In nine of the patients in Group_INNER_, ${\varepsilon}_{\mathrm{mean}}^{\mathrm{rel}}$ was lower than −5% in the lower tongue. This was due to the artifact region partly extending into the area of the ROI, resulting in subregions with large relative differences.

The size of the artifacts of the patients in Group_OUTER_ increased compared to the artifacts of Group_INNER_. The resulting bias in AC-PET was severe in regions in and near the signal voids (Figure 4H). Note the similarity of tracer distribution of Figure 4F,G near and in the signal void suggesting that our method has recovered the PET signal with minimal bias.

Of note, the bias is present also in areas further away from the implants in Group_OUTER_ (cerebellum, ${\varepsilon}_{\mathrm{mean}}^{\mathrm{rel}}$ −1.8%, range, −7% to 0%; ${\varepsilon}_{\max}^{\mathrm{rel}}$ −1.8%, range, −11% to 2.4%). In selected cases, this bias may markedly affect regions used commonly as reference regions in kinetic modeling. In addition, the kinetic modeling will be biased even with artifact correction in place because of the systematic underestimation and radial bias of the tissue activity concentration from standard MR-AC due to the lack of bone [29].

Interestingly, a positive bias is observed in regions near the artifacts (Figure 7). As hypothesized in [30], this effect could be due to scatter correction. However, this was investigated using reconstructions with and without scatter correction, and the same positive bias was observed. We, therefore, conclude that this effect could be related to the impact of an erroneous attenuation correction on the OSEM reconstruction algorithm, a subject that could be further analyzed. The size of the positive bias is dependent on the size of the artifact. The positive bias was mainly present in regions with low tracer uptake. There was a maximum relative change of 35% in the left masticatory muscle in the sample Group_OUTER_ patient, but the absolute change was only 0.1 SUV in that specific voxel. The positive bias was mainly observed in the ROI placed in the masticatory muscles but was observed also in the lower tongue with a positive bias of 10% in a single patient as this ROI was placed directly in-between a bilateral dental artifact in an area with low tracer uptake (${\varepsilon}_{\mathrm{mean}}^{\mathrm{abs}}$ 0.14 SUV).

**Figure 9:**
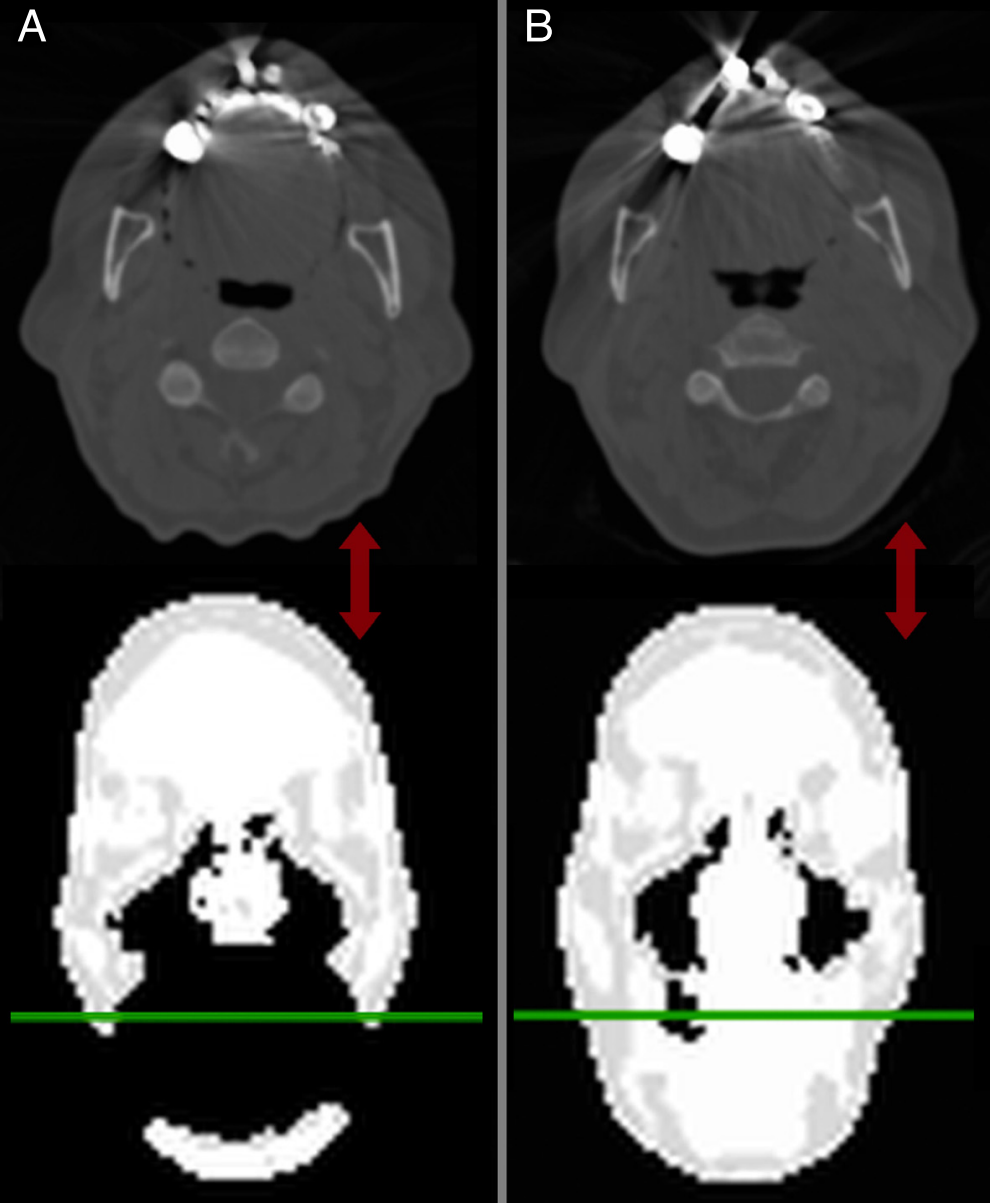
**Demonstration of the irreproducibility of the artifact sizes between scans.** MR-AC (bottom row) compared to CT (upper row). **(A,B)** MR-AC_DIXON_ and CT images of the same patient acquired 4 months apart. Note that the magnitude and size of CT artifacts seem unchanged between the scans.

The metal artifacts are usually less severe in the CT images than in the MR-AC maps, since the number of voxels affected by the metal implant is much larger on MR than the actual implant (Figure 3A,B) [12]. Previous studies by Pauchard [11] have shown MR distortions to be dependent on the size and orientation of the implants with respect to the gradient field, so the overall artifact size cannot be predicted from the knowledge of the size of the implant as such. For example, two CT images from a PET/CT study of the same patient acquired 4 months apart suggest that the amount and location of the metal fillings is unchanged between the examinations since the streak artifacts are similar and the number of fillings is the same (Figure 9). However, the attenuation maps from the PET/MR scan of the same patient at the same two-time points were very different and the size of the susceptibility-induced artifacts varied. This is a problem that can have severe implications for follow-up examinations, which can be solved with our inpainting correction method.

The clinical interpretation of the tracer uptake patterns on AC-PET images is difficult in patients with large metal-induced artifacts. In one patient, the tracer uptake increased 19% after correction in a VOI with 50% isocontour levels defined encompassing an entire tumor, and this tumor was located outside the area spanned by the artifact (Figure 8). A tumor located inside the artifact area could potentially have a largely decreased contrast with possible implications for the clinical reading of the attenuation-corrected PET image as a result of the artifact as well as on the MR sequences.

This study has some limitations. First, the absence of a reference value for the correct attenuation map was addressed by visual inspection of each of the MR-AC_INPAINTED_ maps to ensure that the inpainting was done correctly. Furthermore, we manually corrected the artifacts in four patients from Group_INNER_ and seven patients from Group_OUTER_, and by comparing the MR-AC maps and their resulting AC-PET images, we found that our automatic correction method produced very similar results. However, despite careful inspection of the attenuation maps, it is not possible to determine the accuracy of the reported improvements without the reference data, as residual errors introduced by the inpainting algorithm might affect the PET uptake. Further investigations using phantom data or simulated artifacts are required to assess the absolute bias.

Second, the ROI delineation was done partly on the anatomical MR images, which in some cases had severe signal voids. The correct delineation of the ROIs tongue and lower tongue was, therefore, challenged in 10% of the patients, as we had to draw the ROI on the corrected PET_INPAINTED_ image instead. Third, we did not take the attenuation of bone and metal into account. Thus, the correction method outlined is an improvement of the quantitative measures of tissue activity concentration that will improve the clinical evaluation; but compared to a true AC estimates, there will still be a residual error. A related study where the much larger metal endoprosthesis was included only showed local differences to soft tissue only corrections [12], which leads to the conclusion that the overall effect would be minimal.

## Conclusions

Metallic dental work causes severe signal voids on MR images. Subsequent artifacts on MR-AC attenuation maps and AC-PET images may exceed the actual fillings in size. The resulting bias in AC-PET is severe in regions in and near the signal voids. The bias depends on the size and location of the artifacts; in the patients with the largest type of artifacts, the bias is present also in areas further away from the dental fillings. As a consequence, the lesion uptake will be highly reduced inside or near the artifact (Figure 8) and could potentially be obliterated completely. The artifact regions on the attenuation maps can be assigned soft tissue attenuation values as a means of reducing artifact severity, as suggested in [12], but further work is necessary to confirm the absence of bias. Air-only (PET_DIXON_) representations cannot be considered appropriate for clinical applications.

## Additional file

Additional file 1: Figure S1. ROI delineation. (A) sagittal image showing the placement of the manually drawn ROIs: (B) 2D spherical ROI in the lower tongue (cyan), (C) 2D spherical ROI in the masticatory muscles (blue) and tongue (green), (D) 2D spherical ROI in the cerebellum (red).
